# How to detect a polytrauma patient at risk of complications: A validation and database analysis of four published scales

**DOI:** 10.1371/journal.pone.0228082

**Published:** 2020-01-24

**Authors:** Sascha Halvachizadeh, Larissa Baradaran, Paolo Cinelli, Roman Pfeifer, Kai Sprengel, Hans-Christoph Pape

**Affiliations:** 1 Department of Trauma, UniversitätsSpital Zürich, Zürich, Switzerland; 2 Harald Tscherne Laboratory, Department of Trauma, University Zurich, University Hospital Zurich, Zurich, Switzerland; John Hunter Hospital and University of Newcastle, AUSTRALIA

## Abstract

**Introduction:**

Early accurate assessment of the clinical status of severely injured patients is crucial for guiding the surgical treatment strategy. Several scales are available to differentiate between risk categories. They vary between expert recommendations and scores developed on the basis of patient data (level II). We compared four established scoring systems in regard to their predictive abilities for early (e.g., hemorrhage-induced mortality) versus late (Multiple Organ Failure (MOF), sepsis, late death) in-hospital complications.

**Methods:**

A database from a level I trauma center was used. The inclusion criteria implied an injury severity score (ISS) of ≥16 points, primary admission, and a complete data set from admission to hospital-day 21. The following four scales were tested: the clinical grading scale (CGS; covers acidosis, shock, coagulation, and soft tissue injuries), the modified clinical grading scale (mCGS; covers CGS with modifications), the polytrauma grading score (PTGS; covers shock, coagulation, and ISS), and the early appropriate care protocol (EAC; covers acid–base changes). Admission values were selected from each scale and the following endpoints were compared: mortality, pneumonia, sepsis, death from hemorrhagic shock, and multiple organ failure.

**Statistics:**

Shapiro-Wilk test for normal distribution, Pearson Chi square, odds ratios (OR) for all endpoints, 95% confidence intervals. Fitted, generalized linear models were used for prediction analysis. Krippendorff was used for comparison of CGS and mCGS. Alpha set at 0.05.

**Results:**

In total, 3668 severely injured patients were included (mean age, 45.8±20 years; mean ISS, 28.2±15.1 points; incidence of pneumonia, 19.0%; incidence of sepsis, 14.9%; death from hem. shock, 4.1%; death from multiple organ failure (MOF), 1.9%; mortality rate, 26.8%). Our data show distinct differences in the prediction of complications, including mortality, for these scores (OR ranging from 0.5 to 9.1). The PTGS demonstrated the highest predictive value for any late complication (OR = 2.0), sepsis (OR = 2.6, p = 0.05), or pneumonia (OR = 2.0, p = 0.2). The EAC demonstrated good prediction for hemorrhage-induced early mortality (OR = 7.1, p<0.0001), but did not predict late complications (sepsis, OR = 0.8 and p = 0.52; pneumonia, OR = 1.1 and p = 0.7) CGS and mCGS are not comparable and should not be used interchangeably (Krippendorff α = 0.045).

**Conclusion:**

Our data show that prediction of complications is more precise after using values that covers different physiological systems (coagulation, hemorrhage, acid–base changes, and soft tissue damage) when compared with using values of only one physiological system (e.g., acidosis). When acid–base changes alone were tested in terms of complications, they were predictive of complications within 72 hours but failed to predict late complications. These findings should be considered when performing early assessment of trauma patients or for the development of new scores.

## Introduction

Early assessment of the clinical status of severely injured patients is of pivotal importance in guiding surgical and intensive care management [1–4]. Blood transfusions have been associated with acute and long-term complications [5–9]. Most authors agree that the prediction of early mortality is equally important as predicting complications in the later stages.

Recently, the initial, elevated lactate level value gained more attention for early assessment of trauma patients. Moreover, this value was to be relevant in predicting early complications (24-hour mortality) [6]. Dezman et al. [7] stated that the underlying population of trauma patients includes a fair number of penetrating injuries known to result in acute, sustained blood loss. In a similar population, the same group identified “failure of lactate clearance” as an important predictor of 24-hour mortality [7]. Late complications have not been addressed by these authors [7, 8].

Coagulopathy alone is known to represent a relevant guide for treatment and for the prediction of complications, especially those that occur in the later clinical course [8, 9]. Likewise, coagulopathy is known to be related to acute hemorrhage and the requirement of mass transfusion protocols. It has been associated with delayed resuscitation and reperfusion injury [10] and soft tissue injury [11]. Especially in patients with coagulopathy or elevated lactate levels, treatment recommendations have been made to address these principles [7]. The available guidelines have attempted to guide the management of orthopedic injuries within the first days after trauma. However, it is unclear if these principles are relevant in a general trauma population are equally relevant for patients with orthopedic injuries [7]. Although our group has recently shown improved outcomes after changes of transfusion protocols were made, it is unclear if these changes are relevant in comparison to other pathogenetic changes [12].

To our knowledge, no study has compared the relevance of published studies involving parameters covering several pathways, such as coagulopathy, acidosis, and the additional effects of detected soft tissue injuries in a separate database. Our study addresses this issue and compares existing treatment recommendations to answer the following questions:

- Can early clinical assessment in multiple injured patients predict both early and late complications?
- Do recommendations based on multiple pathways (e.g., shock, acidosis, and coagulopathy) reliably predict complication rates in multiple injured patients?
- Are laboratory parameters from a single pathway equally predictive as those described in a multiple pathway approach?

## Methods

### Database and study population

A prospective database that encompasses all clinical parameters (parameters relevant for emergency room treatment, initial operative strategy, intensive care stay, and in-hospital complications) was used. This database includes multiple injured patients treated at a Level 1 trauma center from January 1, 1996 to January 1, 2013. The study population had to fulfil the following inclusion criteria: adult patients, treated due to polytrauma at one Level 1 trauma center, and an admission time of less than 24 hours after the trauma. Patients with oncological diseases, chronic diseases, and genetic disorders that affect the musculoskeletal system were excluded. All data were retrieved from patient records, as approved by the local institutional review board (IRB) Swissethics Kantonale Ethikkommision Züruch (KEK-Zürich), according to the University of Zurich IRB guidelines, and the study was conducted according to the guidelines of good clinical practice (“Retrospektive Analysen in der Chirurgischen Intensivmedizin” Nr. StV. 01–2008). The ethical committee waived the need for consent. In the database, the trauma physician that routinely performs the scoring of injury severity classifies all injuries. Data include twice-daily entries of clinical and physiological parameters and organ function scores during the first three weeks of admission [13, 14]. The database covers all parameters including clinical chemistry, hemostasis, and ventilation-associated parameters during the first weeks of hospitalization. Admission data were only required in the emergency room, whereas all additional laboratory data were acquired on a daily basis. Laboratory data were collected from admission to 21 days after trauma. All outcome events (early and late complications) were recorded in a longitudinal manner [15]. Patients were scored strictly based on the recommendation of each scoring system based on measurements and values available at the end of the ER diagnostics.

### Definitions

The severity of the injury was graded according to the injury severity score (ISS) [16] and evaluated based on the information available at discharge. Major fractures include fractures of the femur, the tibia, the spine, and the pelvis. Polytrauma patients were defined as having an ISS greater than or equal to 16, along with the criteria of the Berlin definition [17]. Thoracic and abdominal trauma were classified according to their abbreviated injury scale (AIS) [18, 19]. Lung contusion was independently diagnosed by a radiologist. The severity of a head injury was graded according to the Glasgow coma scale (GCS) [20].

### Scoring systems

Six published recommendations were evaluated regarding their usability for preoperative decision-making. One scoring system found that a 48-hour ventilation period was predictive of complications [21]. This parameter was not deemed feasible for clinical use prior to surgery, and the score was excluded. Clinical unfeasibility applied further to a score used to assess the risk of massive transfusion [22]. Therefore, the current study included four different published recommendations to stratify and categorize multiple injured patients in our database. Patients were stratified according to the early appropriate care (EAC) protocol strictly using the recommendations of the authors [23]. Furthermore, stratification according to the modified clinical grading system (mCGS) was strictly performed, as described by the authors, including the assessment of the number of packed red blood cells (pRBCs) within 24 hours [23]. This modified score based on the CGS was also used to stratify our patients according to the recommendations of the authors. [24]. Here, D-dimer levels, urinary output, the thoracic trauma score, and the oxygen ratio (PaO_2_/FiO_2_) were omitted for the current study. Also, the PTGS was evaluated [25].

### Clinical grading system (CGS)

The CGS [24] represents a summary of multiple publications and lists parameters indicative of four different pathogenic pathways. Its level of evidence is based on expert knowledge (level IV) and has not been validated in a database. All recommendations rely on studies prior to 2005. In comparison with the mCGS (see below), factor V, fibrinogen, platelet count, and the advanced trauma life support (ATLS) classification were included.

### Modified clinical grading system (mCGS)

The mCGS [23] represents a modification of the recommendations from 2005 that encompassed the following changes: parameters omitted from the CGS included factor II and V, fibrinogen, D-dimer, ATLS classification, urine output, PaO_2_/FIO_2_, and thoracic trauma score due to limitations in data availability. The transfusion parameter was modified from blood units (2 hours) to number of pRBCs transfused on the day of injury. Suggested parameter values associated with all criteria except platelet count overlapped and were modified so that patients could be clearly assigned to a clinical grade. This was applied, in particular, to the number of pRBCs transfused (from blood units within 2 hours after admission (CGS) versus blood units administered within 24 hours after injury (mCGS)), as it was felt that these changes modify the meaning of the scale [26].

### Early appropriate care (EAC) protocol

The EAC [23] protocol was developed based on data from 1443 adult patients treated between 1999 and 2006 in a level I trauma center. The mean ISS was 24.7 (range, 9–57), thus including isolated fractures. The aim of the development of the EAC was to facilitate the clearance of patients for definitive surgeries by orthopedic surgeons, after assessment by general surgery and neurosurgical clearance. The database encompassed fractures of the pelvis (n = 291), acetabulum (n = 399), spine (n = 102), and/or the proximal or femur shaft (n = 851). The EAC foresees the use of lactate, pH, and base excess and utilizes a dichotomous approach that distinguishes between low-risk and high-risk patients. According to the authors, definitive surgery of all of these fractures is recommended when patients fall into the low-risk group. It is important to note that there were patients who had provisional external fixation of their femur or pelvis followed by later conversion to internal techniques. These patients were grouped into the late fixation group.

### Polytrauma grading score (PTGS)

The PTGS was calculated on the basis of a nationwide trauma registry [25]. The results were calculated from data of 11,436 multiply-injured patients treated in multiple trauma centers between 1994 and 2012. The following inclusion criteria were applied: age, >16 years; AIS, ≥3 points and treatment in an intensive care unit; or ISS ≥16 points. None of these patients had isolated major fractures. Addendum 1 summarizes the required values and used values to stratify patients according to the included scoring systems.

### Outcome parameter and endpoints

Outcome parameter include multiple organ failure (MOF), as previously described [27], acute respiratory distress syndrome (ARDS) [28], pneumonia (temperature, ≥38.5°C; radiologic signs of infiltration; absence of ARDS), sepsis (temperature, ≥39.0°C; central–peripheral temperature difference greater than 8°C; positive fluid balance, +1500 mL/24 h; and leukocyte count, <4,000 or >12,000/μL), and mortality. Mortality was distinguished by death within 72 hours, death due to traumatic brain injury (TBI), death due to hemorrhage, or death due to MOF. The diagnosis of infection was made for every soft tissue change that lead to a combination of redness, swelling, and development of drainage, requiring surgical or pharmacological intervention [29].

Early complications included death within 72 hours, death from TBI, and death from exsanguination. Late complications included pneumonia, sepsis, and death from MOF. The acid–base system includes pH, lactate, and base excess values. Coagulation includes measurements of platelet count, fibrinogen, and prothrombin time. Hemorrhage includes systolic blood pressure and the number of pRBCs within 2 hours of admission. Soft tissue includes measurement of the severity of thoracic trauma [18], abbreviated injury scale for integument, and the Moore classification for severity of intra-abdominal injury [30].

### Application and validation of scoring systems in a comparative dataset

The prediction of all of these scoring systems towards the aforementioned outcome parameters was tested. All scoring systems were applied to all patients. The prognostic ability of each score was calculated for every subcategory, and patients were stratified according to the scoring recommendation. A categorization within the scores, according to complication and mortality, was performed. The results were weighed according to the risk of complications. CGS and mCGS were compared directly since validation of the EAC is based on the mCGS [23] in order to assess its validity. For comparison of the mCGS and CGS, we used complete datasets of patients for these variables. Those patients were stratified according to mCGS and CGS. The agreement of strata was further evaluated based on Krippendorff’s alpha reliability estimate.

### Statistics

Nominally-scaled and dichotomous variables were compared with the Pearson Chi-square test. Fitted, generalized linear models were used for predictive estimates of the scoring systems. OR for the prognosis of the different endpoints were calculated, along with 95% confidence intervals (CIs). All tests were corrected for multiple testing if necessary. Proportions were evaluated using the Yates-corrected statistics. The relative risks of complications were calculated individually and expressed in ORs. Prediction model bases on dichotomous outcome variables and continuous measurements. Prediction models were based on generalized, linear, mixed-model analysis and are displayed as Receiver Operating Curve (ROC) curves with Area under the Curve (AUC) and 95% CI. The associations between conventional parameters and death were evaluated using univariate analysis. Continuous variables were summarized as means and standard deviations. Statistical significance was set at a p-value of <0.05. All calculations were performed using R Core Team (2018) (R: A language and environment for statistical computing, R Foundation for Statistical Computing, Vienna, Austria, URL: https://www.R-project.org/).

## Results

This study included 3668 patients for stratification, according to the included scoring systems. The mean ISS was 28.2 points (±15.1 points), the mortality rate was 26.8%, and the overall complication rate was 24.7%. The mean age at time of injury was 45.8 years (±20.2 years). The demographics of the included patients are summarized in Table 1.

**Table 1:** Demographics and outcome parameters.

N = 3668	Mean ± SD	Median
Age at injury (years)	45.8 ± 20.2	44
Glasgow coma scale (GCS)	8.8 ± 5.5	10
Length of hospital stay (days)	17.0 ± 18.7	13
Length of intensive care unit stay (ICU, days)	8.2 ± 10.5	4
Duration of ventilatory support (days)	5.1 ± 8.1	1
ISS	28.2 ± 15.1	25
NISS	37.2 ± 17.4	34
All complications	24.7%	
Pneumonia	19.0%	
Sepsis	14.9%	
Bacteraemia	7.9%	
Septic Shock	3.2%	
Mortality	26.8%	

SD: Standard Deviation

ISS: Injury Severity Score

NISS: New Injury Severity Score

### Score evaluation

Following the application of data availability for a given score and exclusion of patients that succumbed during treatment in the emergency room, 3026 (82.5%) patients were stratified according to the EAC, 2155 (58.9%) were stratified according to the mCGS, 2246 (61.2%) were stratified according to the CGS, and 2193 (59.8%) were stratified according to the PTGS. All scales demonstrated an increase in mortality between the lowest and the highest graded value, from about 21.9% (stable/low-risk patients) to 66.7% (in extremis/high-risk patients). Furthermore, the risk for early death (within 72 hours) increased significantly the less stable the patients were (Table 2).

**Table 2:** Predictive capability of each score individually compared to the lowest scoring grade.

Complication	Score	Scoring Strata	OR	95% CI	P-value
Pneumonia	EAC	High Risk	1.1	0.8–1.5	0.74
	mCGS	Unstable	0.9	0.7–1.1	0.31
		Borderline	0.7	0.5–1.0	0.06
		In Extremis	0.4	0.1–2.0	0.29
	CGS	Unstable	0.9	0.7–1.2	0.48
		Borderline	0.8	0.6–1.0	0.09
		In Extremis	0.6	0.2–1.6	0.31
	PTGS	Unstable	1.5	1.1–1.9	0.01
		Borderline	1.3	0.6–2.8	0.48
Sepsis	EAC	High Risk	1.1	0.8–1.5	0.50
	mCGS	Unstable	0.9	0.7–1.2	0.42
		Borderline	0.9	0.7–1.3	0.62
		In Extremis	0.3	0.0–2.2	0.23
	CGS	Unstable	1.0	0.8–1.3	0.87
		Borderline	1.0	0.7–1.3	0.91
		In Extremis	0.7	0.2–1.9	0.47
	PTGS	Unstable	1.5	1.2–2.0	0.00
		Borderline	1.8	0.9–3.6	0.09
Death from MOF					
	EAC	High Risk	2.1	1.0–4.1	0.04
	mCGS	Unstable	0.7	0.4–1.4	0.33
		Borderline	0.5	0.2–1.5	0.24
		In Extremis	5.6	1.2–25.8	0.03
	CGS	Unstable	0.5	0.3–1.0	0.04
		Borderline	0.3	0.1–0.8	0.02
		In Extremis	2.9	0.8–10.1	0.09
	PTGS	Unstable	2.2	1.2–4.1	0.02
		Borderline	4.2	1.2–14.2	0.02
Death within 72 hours	EAC	High Risk	1.5	1.4–1.6	<0.001
	mCGS	Unstable	1.1	1.0–1.1	0.001
		Borderline	1.2	1.1–1.2	<0.001
		In Extremis	1.4	1.2–1.7	<0.001
	CGS	Unstable	1.0	1.0–1.1	0.021
		Borderline	1.2	1.1–1.2	<0.001
		In Extremis	1.4	1.2–1.6	<0.001
	PTGS	Unstable	1.1	1.1–1.2	<0.001
		Borderline	1.3	1.2–1.5	<0.001

Odds Ratio (OR) are in referenced to low risk (in case of EAC) and to stable (all other scores) patients within each score. With increase instability, the risk of death within 72 hours increases significantly. This leads to patients, that initially were stratified to as borderline, or in extremis that die prior to the development of late complications (Pneumonia, Sepsis, or death due to MOF)

EAC = Early Appropriate Care

(m)CGS = (modified) Clinical Grading System

PTGS = Polytrauma Grading Score

### Comparability of mCGS and CGS

The mCGS shows sustained changes regarding prediction of complications when compared to the original recommendation (CGS). When comparing the agreement of stratification, the Krippendorff analysis revealed α = 0.04. Our results demonstrate that the mCGS grades patients towards more stable conditions compared to CGS. This shift is unidirectional since no patient was graded as more stable by the CGS compared to the mCGS (Table 3).

**Table 3:** Changes in patient risk assessment by modification of the CGS to the mCGS.

		**CGS**			
		Stable	Borderline	Unstable	In Extremis
**mCGS**	Stable	757 (35.1%)	193 (8.9%)	9 (0.4%)	1 (0.05%)
	Borderline	0	726 (33.7%)	107 (5.0%)	1 (0.05%)
	Unstable	0	0	331 (15.4%)	12 (0.6%)
	In Extremis	0	0	0	18 (0.8%)

The agreement of CGS and mCGS was assessed with the Krippendorff analysis (α = 0.0459)

### Strata of patients according to the initial assessment

The EAC protocol separates low-risk and high-risk stratified patients and does not stratify patients into borderline or unstable conditions.

Our results reveal a high sensitivity for early death and hemorrhagic shock, but no predictive abilities of the EAC for late complications, such as pneumonia, sepsis, or infections (Table 4).

**Table 4:** Ability to predict early (within 72hours) versus late (after 72hours) complications in patients classified according to EAC.

		Low Risk	High Risk	Pearson χ^2^
		n = 2745	n = 281	p-value
Early Complication	Total Mortality	22.3%	61.2%	<0.0001
	Death within 72h	14.2%	56.2%	<0.0001
	Death from TBI	17.5%	25.9%	0.0006
	Death from exsanguination	1.2%	27.0%	<0.0001
	Infection	31.3%	27.4%	ns
Late Complication	Death later 72h	8.1%	5.3%	ns
	Pneumonia	19.9%	20.9%	ns
	Sepsis	15.9%	17.4%	ns
	Bacteraemia	7.9%	10.2%	ns
	Septic Shock	25.6%	5.6%	ns
	Death due to MOF	1.7%	3.5%	ns

ns: not significant

TBI: Traumatic Brain Injury

MOF: Multiple Organ Failure

When patients were stratified as “Low Risk” according to the EAC, 40.3% of these patients were still categorized as not stable according to the CGS; 23.6% were stratified as stable, 27.7% were stratified as borderline, 11.8% were stratified as unstable, and 0.8% were stratified as “in extremis”. Albeit, stable, stratified patients seemingly suffer from more complications (e.g., pneumonia), and unstable patients had a higher risk of mortality within 72 hours (i.e., deceased prior to developing complications). Patients that were assessed based on acid–base changes alone (“high-risk” patients according to the EAC) showed sustained changes in complications or mortality rates when stratified according to pathological values of other functional systems (e.g., hemorrhage or coagulation). Patients graded as “high risk” according to the EAC were indicated to have pathological values of the acid–base system. A stratification of these patients according to pathological values of the hemorrhagic system triples the risk of mortality (OR 3.1, 95% CI: 1.6–5.9, p<0.0001) compared to those patients without pathological values of the hemorrhagic system. When stratifying according to soft tissue injury, the risk of developing sepsis nearly quadruples (OR 3.8, 95% CI: 1.1–12.8, p = 0.021), compared to patients without soft tissue injury within the “high risk” patients.

### Prediction of complications

**Fig 1:**
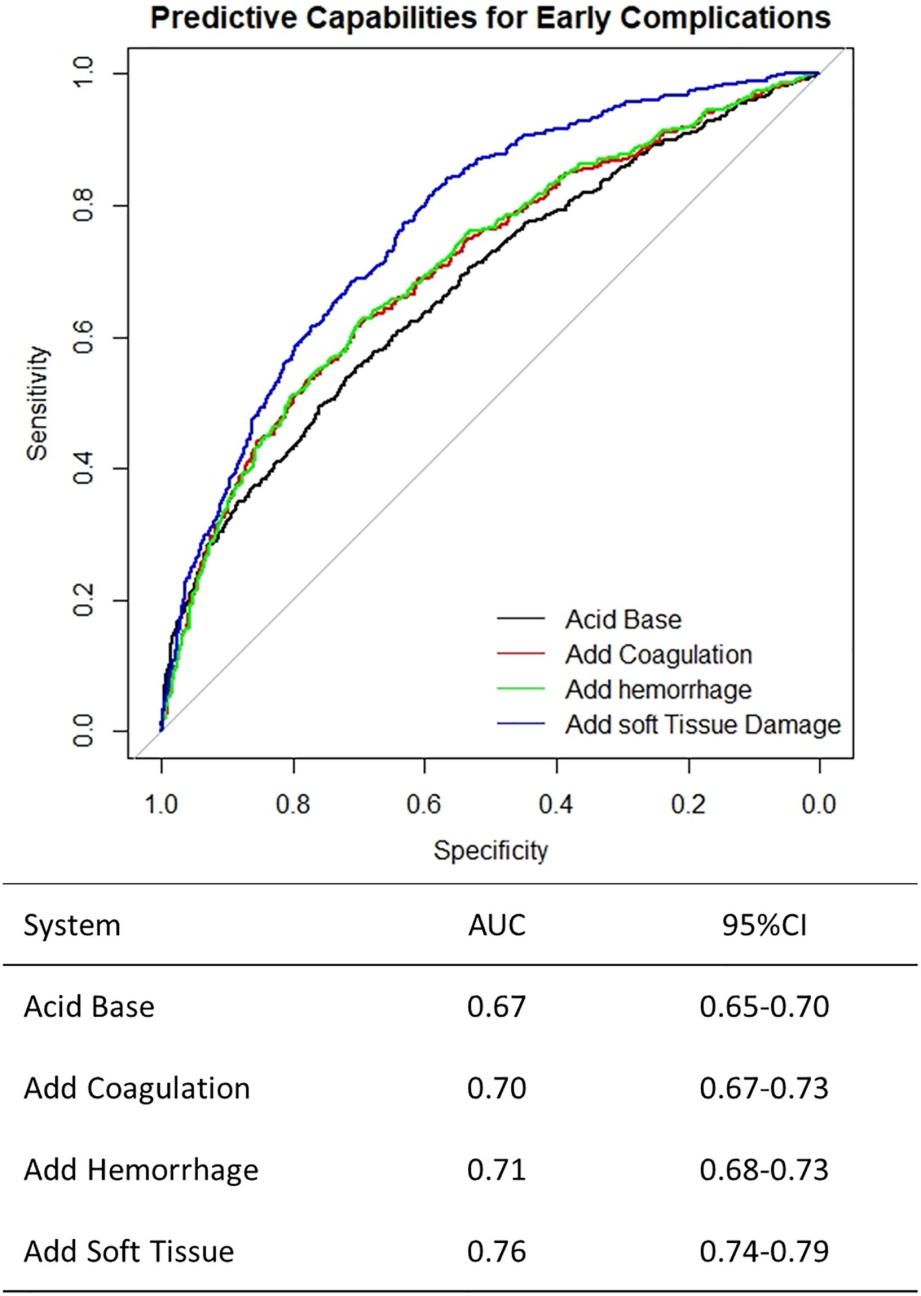
Comparison of the ability to predict early complications of acid base changes alone (black), addition of coagulopathy (red), addition of acute hemorrhage (green), and addition of soft tissue injuries (blue). The addition of these parameters lead to a sustained improvement in prediction of complications. Early Complications include Death within 72 hours, Death from traumatic brain injury, Death from exsanguination. AUC = Area Under the Curve. 95%CI = 95% Confidence Interval.

We found that the combination of measurements of acid–base changes, coagulation, hemorrhage, and severity of soft tissue injury increase predictive capabilities for complications. The prediction model that used measurements only from the acid-base system (Lactate, BE, pH) yields an AUC of 0.67 (95%CI: 0.65–0.7). However, when measures of the coagulation system (PT, Platelet, Fibrinogen etc.) were added to the prediction mode, the AUC increases to 0.70 (95% CI: 0.67–0.73). Combining measures of all systems (acid-base, coagulation, hemorrhage, and soft tissue damage) yielded the highest AUC (0.76, 95%CI: 0.74–0.79, Fig 1).

## Discussion

Polytrauma accounts for approximately 5.8 million deaths worldwide [31, 32]. Although the clinical development of each patient may vary substantially depending on injury distribution (e.g., truncal versus head injuries), the prevention of late in-hospital complications, such as MOF and sepsis, is crucial [33].

In this line, scoring systems may be helpful in predicting the risk of complications. Most of the available scores rely on anatomical, physiological, and biochemical parameters, or a combination of these [34, 35]. While several scores were found to be relevant for preclinical use, those applicable during the early in-hospital stay appear to be sparse and they focus on parameters of hemorrhagic shock [23, 36], or predict massive transfusion [22]. To our knowledge, six different grading recommendations are currently available, among which, two represent level IV evidence and three utilize a database to develop their score [22–25, 37, 38]. Other recent publications also utilize massive transfusion as the main predictor for complications, but they do not provide treatment recommendations [22, 39].

For the current study, we were aware of the following drawbacks and strengths:

First, the selection of parameters in the coagulation pathway may be criticized. Platelet count, as used in the CGS and the mCGS, or International Normalized Ratio (INR), as used in the PTGS, may be of limited relevance since new methods are available [40–42]. Second, we included published recommendations that only reach level IV evidence. However, according to our literature search, only six publications have become available within the last three decades that describe prediction based on routine clinical parameters. Of these, two [22, 43] used parameters that may not be readily available or they included ventilation for >48 hours and were not deemed to be feasible for comparison. Of those included in this study, two used a database to develop their recommendations [23, 25], but they did not provide a validation group to support their assumptions. Nevertheless, they both utilized the previous level IV recommendation (CGS, mCGS) to test their hypothesis and graded the patient according to the clinical conditions (stable, borderline, unstable, in extremis). Additionally, they described treatment recommendations based on their predictive degrees. One study modified the first level IV recommendation due to a lack of availability in their database and criticized its value while using the modified recommendation. This modification represents a downgrading of the severity of injury, which may be relevant for polytrauma patients [44].

Third, inclusion of the PTGS may be subject to criticism since it excludes patients categorized as “in extremis” by definition. The threshold levels for this score appear to be rather high, which may explain the higher mortality rates in the PTGS after application of all other scores. However, since it represented one of the two scores developed in the database analysis and the number of patients was relevant (11,438 versus 1443 in the EAC), its validity is favorable. The size of our database may be a strength because it encompasses one of the largest data collections of a single Level 1 trauma center currently available. In comparison, Regel summarized 3406 patients over a period of about 20 years and described distinct complications [1]. The inflammation and the Host Response to Injury Large-Scale Collaborative Program included 1,537 patients from seven trauma centers over eight years [45].

Firth, our rationale to include patients treated before the change of the millennium (1996–2000) and to discontinue including patients after 2013 may be criticized. However, this decision was made for the following reasons:

1. In order to compare scoring systems, we aimed for inclusion of patients from similar periods as the most recent publications [23, 25].
2. In addition, we tested whether inclusion of patients before 2000 would alter the results, which was not the case.
3. A new transfusion protocol has been introduced in 2014 [12, 46] and was associated with a substantial improvement in outcome. We therefor excluded patients that were treated later than 2013. Since the mCGS and CGS include the transfusion protocol as a variable, we believe that the new transfusion protocol would substantially change the score results.

Fifth, the issue of missing values in this study as seen in Table 1 has to be considered. We agree, that ideally the results should have been derived from an identical dataset for all classification systems. However, in our attempt to aim for high power, we selected the maximum available dataset.

We feel that our results are reliable enough to support our main conclusions. In general, categorization into different risk groups revealed a comparable distribution in mortality rates between stable/low-risk (21.9–25.4%) patients and in extremist/high-risk (60.0–66.7%) patients throughout all four scores. However, we found sustained differences between the four scores, as follows:

1. Comparison of the CGS and mCGS revealed that the modification leads to sustained differences in the outcomes when applying both scores. The agreement analysis revealed that the CGS graded more patients as borderline, unstable, and in extremis compared to mCGS, thus, a interchangeable use of these grading systems is not recommended.
2. Patients graded as borderline according to the PTGS demonstrated a higher mortality rate (50%) compared to those graded as borderline according to the CGS (35.9%) or mCGS (37.8%).
3. The EAC revealed significant predictive differences between low-risk and high-risk patients for shock-related complications (death from hemorrhage, death within 72 hours), but no differences were revealed for those occurring later during hospitalization.
4. Adding measurements from other physiological systems (e.g., coagulation, hemorrhage, soft tissue injury) to measures of acid–base changes improves prediction of complications substantially.

With regard to our first finding, the differences between both scoring systems lie in the choice of parameters selected for modification, which have been discussed previously. Two of the modifications appeared to be most relevant for the observed changes. The classification of chest injuries was different in the mCGS since patients without chest trauma were also included. This may lower the degree of chest injury in this particular patient group, thus affecting the risk of complications [47].

The CGS includes the numbers of pRBCs administered within the first two hours, whereas the mCGS uses the number administered within 24 hours. The treating surgeon should consider these differences when determining the clearance of patients for surgery.

The development of the PTGS aimed to improve the definition of the “borderline” patient. It appears that the rate of complications differ substantially when patients are stratified according to PTGS compared to other scores. This difference is based on the fact, that the threshold levels used in PTGS for “borderline (admission blood pressure of <75 mmHg, a New Injury Severity Score (NISS) of >50 points, or a pRBC of 15 or more) resemble patients stratified as “in extremist”, according to other scores. This may be due to the availability of data in the registry. Certain measurements of the acid–base system, or the coagulation system are not included in the registry. Also, while the data base encompasses a large number of patients, variables from two categories (base deficit and body temperature) were only available in half or less of the patients. In the subgroup analysis for separation of low, intermediate, and high mortality, no patient data were found for platelet count and temperature, which may represent a systemic bias. Finally, due to the exclusion of patients stratified as “in extremis” in the development of the dataset, sicker patients from the original registry might have resulted in different values.

Regarding our third finding of the patients grouped according to the EAC, the vast majority of them were grouped as “low risk”. As discussed above, acid–base changes can normalize rapidly, which may lead to a selection of patients that are apparently low risk but have sustained injuries that may put them at risk of later complications. When comparing low-risk and high-risk groups, significant differences were found only the rate of short-term complications. The overall best predictive ability was 70% for death from acute hemorrhage. In contrast, the rate of late complications (those occurring later than 72 hours after injury) were comparable in both strata “low-risk” and “high-risk”. Therefore, we feel that additional information should be available for decision making regarding clearance for major surgery and to prevent unexpected complications. More recently, the importance of coagulopathy has been stressed. Kutcher et al. [45] convincingly demonstrated that coagulopathy has deleterious effects, independent of injury severity, shock, and the “vicious triad”. An additional study from a major Level 1 trauma center reinforced the pivotal importance of the clotting system and described two distinct phenotypes within the entirety of global clotting factor abnormalities [48]. This is in accordance with the recommendations of Inflammation and the Host Response to Injury Collaborative Research Program, which reinforced the relevance of numerous clinical parameters and biomarkers for the prediction of clinical complications. Likewise, the authors stated that post-injury organ failure continues to be a threat [49].

Our results are in line with these findings since the inclusion of several functional systems in any of the scores investigated in the current study was superior to using a single system only. When parameters directly or indirectly indicative of hemorrhage were used, the prediction was focused on early complications related to hemorrhagic shock but not later complications. Clinicians use indicators of the acid–base system routinely as a quick indicator of hemorrhagic shock. If the surgical effort to stop the bleeding is successful, pathologic values of the acid-base system usually recovers within hours [50, 51]. However, soft tissue injuries initiate different pathways that take longer time to normalize. The associated hypoperfusion in severe soft tissue trauma of the pelvis and the extremities is associated with a substantial inflammatory response, as described in multiple studies [50, 52, 53] and clinical settings [22]. Therefore, although shock is an important parameter, it may be more relevant in the prediction of the risk of later complications. These might occur in association with soft tissue injuries and cause prolonged inflammatory stimuli, delayed tissue necrosis, requirement of revision surgeries, and associated organ failure [54].

## Conclusion

In response to the questions addressed in the introduction, we are able to provide the following answers:

Early clinical assessment in multiply-injured patients predicts both early and late complications if the score uses multiple functional pathways (e.g., shock, acidosis, coagulopathy).

Recommendations based on multiple pathways (e.g., shock, acidosis, coagulopathy) reliably predict organ failure and sepsis late after trauma.

Scores that use parameters from a single pathway are less equally predictive than those described in a multi-pathway approach. Pathological acid–base changes predict early mortality but not late complications.

We conclude that among available scales and scores that provide recommendations for orthopedic surgical care in polytrauma patients, those covering multiple pathways are superior to scores that use acid–base changes only. Further clinical use of scoring systems, or new score developments, should, therefore, cover multiple pathways in order to provide adequate predictability of both early and late complications.

## Supporting information

S1 Table (DOCX)
