# Supplementary material for: How to detect a polytrauma patient at risk of complications: A validation and database analysis of four published scales
Source: PLoS One. 2020 Jan 24;15(1):e0228082. doi: 10.1371/journal.pone.0228082 (PMC6980592; doi:10.1371/journal.pone.0228082)
Supplement: S1 Table — (DOCX) [file pone.0228082.s001.docx]

| **Addendum 1: Scoring systems for categorizing injured patients and comparison of systems assessed in score. Numbers or parameters per category** | | | | | | | | |
| --- | --- | --- | --- | --- | --- | --- | --- | --- |
| First Author | Year | Score | Hemorrhage | Acid-Base | Coagulopathy | Temperature | Soft Tissue Injury | Injury Severity |
| Pape | 2005 | CGS | 4 | 2 | 4 | 1 | 6 | - |
| Vallier | 2013 | EAC | - | 3 | - | - | - | - |
| Nahm | 2014 | mCGS | 1+1 | 2 | 1 | 1 | 3 | - |
| Hildebrand | 2014 | PTGS | 2 | 1 | 1 | - | - | 1 |

| **Addendum 2: Distribution of Injury severity according to Abbreviated Injury Scale (AIS)** | |
| --- | --- |
| AIS Head n (%) |  |
| 0 | 850 (23.3) |
| 1 | 262 ( 7.2) |
| 2 | 277 ( 7.6) |
| 3 | 558 (15.3) |
| 4 | 722 (19.8) |
| 5 | 868 (23.8) |
| 6 | 109 ( 3.0) |
| AIS Face n (%) |  |
| 0 | 2625 (73.0) |
| 1 | 219 ( 6.1) |
| 2 | 471 (13.1) |
| 3 | 220 ( 6.1) |
| 4 | 57 ( 1.6) |
| 5 | 2 ( 0.1) |
| AIS Thorax n (%) |  |
| 0 | 1727 (47.6) |
| 1 | 145 ( 4.0) |
| 2 | 188 ( 5.2) |
| 3 | 1042 (28.7) |
| 4 | 362 (10.0) |
| 5 | 163 ( 4.5) |
| 6 | 4 ( 0.1) |
| AIS Abdomen n (%) |  |
| 0 | 2554 (71.0) |
| 1 | 24 ( 0.7) |
| 2 | 237 ( 6.6) |
| 3 | 244 ( 6.8) |
| 4 | 332 ( 9.2) |
| 5 | 203 ( 5.6) |
| 6 | 1 ( 0.0) |
| AIS Spine n (%) |  |
| 0 | 2551 (71.1) |
| 1 | 26 ( 0.7) |
| 2 | 458 (12.8) |
| 3 | 378 (10.5) |
| 4 | 62 ( 1.7) |
| 5 | 109 ( 3.0) |
| 6 | 6 ( 0.2) |
| AIS Extremity n (%) |  |
| 0 | 1715 (47.5) |
| 1 | 181 ( 5.0) |
| 2 | 769 (21.3) |
| 3 | 692 (19.2) |
| 4 | 190 ( 5.3) |
| 5 | 64 ( 1.8) |
| AIS Pelvis n (%) |  |
| 0 | 2870 (80.3) |
| 1 | 26 ( 0.7) |
| 2 | 175 ( 4.9) |
| 3 | 395 (11.0) |
| 4 | 82 ( 2.3) |
| 5 | 27 ( 0.8) |
| AIS Integument n (%) |  |
| 0 | 2225 (62.3) |
| 1 | 878 (24.6) |
| 2 | 384 (10.8) |
| 3 | 58 ( 1.6) |
| 4 | 14 ( 0.4) |
| 5 | 11 ( 0.3) |
